# Neighborhood Broadband and Use of Telehealth Among Older Adults: Cross-sectional Study of National Survey Data Linked With Census Data

**DOI:** 10.2196/26242

**Published:** 2021-06-14

**Authors:** Safiyyah M Okoye, John F Mulcahy, Chanee D Fabius, Julia G Burgdorf, Jennifer L Wolff

**Affiliations:** 1 Department of Health Policy and Management Bloomberg School of Public Health Johns Hopkins University Baltimore, MD United States

**Keywords:** aging, broadband internet, neighborhood, telehealth

## Abstract

**Background:**

The COVID-19 pandemic has amplified the role of telehealth in health care delivery. Regional variation in internet access and telehealth use are well-documented, but the effect of neighborhood factors, including the pervasiveness of broadband internet, on older adults’ telehealth usage in the context of internet access is not known.

**Objective:**

This study aimed to investigate how individual and neighborhood characteristics, including the pervasiveness of neighborhood broadband internet subscription, are associated with engagement in telehealth among older adults with internet access.

**Methods:**

In this cross-sectional study, we included 5117 community-living older adults aged ≥65 years, who participated in the 2017 National Health and Aging Trends Study with census tract–level data for participants’ places of residence from the American Community Survey.

**Results:**

Of an estimated 35.3 million community-living older adults, 21.1 million (59.7%) were internet users, and of this group, more than one-third (35.8%) engaged in telehealth. In a multivariable regression model adjusted for individual- and neighborhood-level factors, age, education, income, and the pervasiveness of neighborhood broadband internet subscription were associated with engagement in telehealth, while race, health, county metropolitan status, and neighborhood social deprivation were not. Among internet users, living in a neighborhood at the lowest (versus highest) tertile of broadband internet subscription was associated with being 40% less likely to engage in telehealth (adjusted odds ratio 0.61, 95% CI 0.42-0.87), all else equal.

**Conclusions:**

Neighborhood broadband internet stands out as a mutable characteristic that is consequential to engagement in telehealth.

## Introduction

The COVID-19 outbreak and the resultant reimbursement and policy changes of the Centers for Medicare and Medicaid Services have motivated a massive shift toward telehealth in routine clinical care [1]. Telehealth refers to the use of communication technologies to deliver health care remotely [2,3]. Older adults are major users of health services but may be unable or unwilling to engage in telehealth owing to the lack of internet-enabled devices, lack of internet access, or difficulties with using technology owing to sensory or functional disabilities or the lack of prior technology experience [2,4]. Older adults also face place-based challenges to telehealth as they are disproportionately likely to live in rural locations that have gaps in internet connectivity, including broadband internet [5,6].

Although the ability to engage in telehealth is affected by both individual and environmental characteristics, data constraints inhibit obtaining knowledge of how such factors jointly contribute to telehealth use. Prior studies have examined individual-level variability in predictors of telehealth use from national surveys, without regard to the neighborhood context [2,7,8]. More recent studies have examined geographic variability in telehealth use among all older adults, which have revealed, for example, smaller increases in primary care telehealth visits among rural providers early during the COVID-19 emergency compared to urban providers [1,4]. This study draws on a unique platform of linked national surveys that together afford comprehensive information on both older adults and neighborhood characteristics to assess the extent to which individual and neighborhood factors may affect engagement in telehealth among older adults with internet access.

## Methods

We conducted a cross-sectional study using data of community-living older adults aged ≥65 years, who participated in the 2017 National Health and Aging Trends Study (NHATS) [9], which were linked to census tract–level data on the participants’ place of residence from the American Community Survey [10]. In the 2017 NHATS, 6312 participants were interviewed: 736 participants lived in residential care facilities or nursing homes and 459 who died were excluded, yielding a final unweighted sample of 5117 participants. Measures of older adults’ sociodemographic characteristics (age, gender, race, education, and income), health status (number of chronic conditions, receipt of help with daily self-care activities, and dementia status), technology use (internet use and engagement in telehealth activities), and county metropolitan status (whether participants lived in a metropolitan or nonmetropolitan county as designated by the US Office of Management and Budget) [11] were drawn from the NHATS. Census-tract measures of broadband internet subscription rates among people aged ≥65 years and a composite measure of neighborhood social deprivation [12] were derived from the American Community Survey and operationalized on the basis of the distribution within the analytic sample by tertile.

As the internet is an important technology for telehealth, we first assessed older adults’ internet usage, defined by self-reports of having browsed the internet on a computer or mobile device in the last month for any reason other than emailing or texting, as previously described [7]. In this study, engagement in telehealth is defined as having browsed the internet in the last year to contact medical providers. Participants who reported browsing the internet in the past month were asked to respond with “Yes” or “No” to the following question: “in the last year have you gone on the internet or online to contact any of your medical providers…this includes making or changing medical appointments, getting test results, requesting referrals or prescriptions, or to get advice?” These activities have been referred to as “digital health technology use” [7] and “health-related internet use” [8] in other studies. We instead refer to these activities as “telehealth” because they have been an important part of the overall massive shift toward web-based health behavior during the COVID-19 emergency [13].

We then comparatively described the characteristics of older adults by internet usage across individual, census-tract, and county characteristics and assessed the adjusted odds of older adults’ internet use in a multivariable logistic regression model that controls for individual, neighborhood, and county characteristics. Furthermore, among the subset of older adults who used the internet, we examined engagement in telehealth. We described the characteristics of older adults who did and those who did not engage in telehealth by individual, census-tract, and county characteristics and assessed the adjusted odds of older adults’ engagement in telehealth in a multivariable logistic regression model that controls for individual, neighborhood, and county characteristics. Reported estimates are weighted with the 2017 survey weights to account for nonresponse, oversampling of subgroups (oldest-old and Black non-Hispanic persons), incomplete interviews, and replenishment of the original sample [14]. Analyses were conducted using Stata (version 13, Stata Corp), and α<.05 indicated significance. NHATS participants provided written informed consent, and our study was approved by the institutional review board of Johns Hopkins Bloomberg School of Public Health (IRB00011232).

## Results

Of an estimated 35.3 million community-living older adults, 21.1 million (59.7%) were characterized as internet users (Table 1). Relative to those who did not use the internet, those who did were younger and more likely to be male, White, more educated, higher income, in better health, and living in metropolitan counties and in census tracts at the top tertile of broadband internet subscription and the bottom tertile of social deprivation, based on unadjusted frequencies. With the exception of the number of chronic conditions and county metropolitan status, these factors were significantly associated with internet use in a fully adjusted multivariable regression model, and women had a significantly higher odds of reporting usage of the internet despite being less represented among internet users.

More than one-third (35.8%) community-living older adults who used the internet in 2017 had engaged in telehealth (Table 2). Internet users who engaged in telehealth were younger (73.5 vs 74.4 years; *P*<.001) and more likely to have a college education (54.0% vs 35.3%; *P*<.001) and be in the highest income tertile (64.4% vs 45.7%; *P*<.001) than those who did not. Internet users who engaged in telehealth were less likely to live in a nonmetropolitan county (11.5% vs 18.3%; *P*=.01) and in neighborhoods at the top 2 tertiles of social deprivation (50.4% vs 62.3%; *P*<.001), while being more likely to live in neighborhoods at the top tertile of broadband subscription (53.7% vs 38.3%).

**Table 1:** Internet usage among community-living older adults.

Parameters		Nonusers^a^ n (weighted %)	Users^a^ n (weighted %)	*P* value^b^	Adjusted odds ratio (95% CI)^c^
Older adults, row (%)		2680 (40.3)	2437 (59.7)	N/A^d^	N/A
Age (years), mean (SE)		78.4 (0.21)	74.0 (0.12)	<.001	0.90 (0.89-0.91)
Females, column (%)		1634 (57.3)	1294 (53.2)	.03	1.32 (1.11-1.58)
Non-White, column (%)		1148 (33.3)	375 (10.2)	<.001	0.31 (0.25-0.39)
**Education**					
	High school or less, column (%)	1798 (66.9)	630 (26.0)	<.001	0.21 (0.16-0.26)
	Some college, column (%)	582 (22.2)	767 (32.0)	N/A	0.48 (0.37-0.64)
	College or beyond, column (%)	300 (10.9)	1040 (42.0)	N/A	Reference
**Income tertiles **					
	<US $27,700, column (%)	1570 (55.2)	477 (18.0)	<.001	0.30 (0.23-0.39)
	US $27,700-$60,000, column (%)	721 (28.8)	751 (29.7)	N/A	0.56 (0.44-0.71)
	>US $60,000, column (%)	389 (16.0)	1209 (52.4)	N/A	Reference
Chronic conditions, mean (SE)^e^		2.67 (0.04)	2.01 (0.03)	<.001	0.95 (0.89-1.02)
Receiving help with daily activities^f^, column (%)		638 (20.4)	203 (7.4)	<.001	0.62 (0.48-0.82)
Dementia, column (%)		528 (15.7)	58 (1.7)	<.001	0.24 (0.17-0.36)
Living in a nonmetropolitan county, column (%)		598 (21.6)	421 (15.9)	.01	0.94 (0.73-1.22)
**Social deprivation index^g^**					
	Lowest tertile (<28), column (%)	441 (18.2)	979 (42.0)	<.001	Reference
	Top 2 tertiles (>28), column (%)	2239 (81.8)	1458 (58.0)	N/A	0.73 (0.56-0.94)
**Broadband subscription^h^**					
	Lowest tertile (<60.9%), column (%)	1403 (47.6)	618 (22.7)	<.001	0.44 (0.33-0.59)
	Middle tertile (60.9%-76.0%), column (%)	769 (32.6)	802 (33.5)	N/A	0.68 (0.52-0.89)
	Highest tertile (>76.0%), column (%)	508 (19.8)	1017 (43.8)	N/A	Reference

^a^Weighted estimates of the 2017 National Health and Aging Trends Study (round 7): 21.1 million nonusers and 14.2 million users.

^b^Pearson chi-square test for frequencies and the adjusted Wald test for means.

^c^Adjusted odds ratios derived from a multivariable logistic regression model that is inclusive of all measures presented in the table.

^d^N/A: not applicable.

^e^Self-reported hearing difficulty, vision difficulty, heart attack, heart disease, hypertension, stroke, hip fracture, diabetes, cancer, lung disease, and arthritis.

^f^Daily activities include eating, bathing, toileting, and dressing.

^g^2015 social deprivation index tertile: ranges from 0 to 100; higher values indicate greater social deprivation.

^h^Broadband subscription rates among older adults in accordance with the 2017 American Community Survey (Table S2802).

**Table 2:** Telehealth use among community-living older adults who use the internet.

Parameters		Nonusers^a^ n (weighted %)	Users^a^ n (weighted %)	*P* value^b^	Adjusted odds ratio (95% CI)^c^
Older adults, row (%)		1608 (64.2)	829 (35.8)	N/A^d^	N/A
Age (years), mean (SE)		74.4 (0.14)	73.5 (0.21)	<.001	0.97 (0.95-0.98)
Females, column (%)		889 (54.6)	405 (50.6)	.12	1.10 (0.87-1.38)
Non-White, column (%)		257 (11.0)	118 (8.7)	.14	0.83 (0.58-1.21)
**Education**					
	High school or less, column (%)	496 (31.2)	134 (16.7)	<.001	0.46 (0.34-0.63)
	Some college, column (%)	528 (33.5)	239 (29.3)	N/A	0.66 (0.52-0.84)
	College or beyond, column (%)	584 (35.3)	456 (54.0)	N/A	Reference
**Income tertiles**					
	<US $27,700, column (%)	384 (22.4)	93 (10.0)	<.001	0.46 (0.33-0.64)
	US $27,700-$60,000, column (%)	527 (31.9)	224 (25.6)	N/A	0.74 (0.56-0.98)
	>US $60,000, column (%)	697 (45.7)	512 (64.4)	N/A	Reference
Chronic conditions, mean (SE)^e^		2.02 (0.5)	1.98 (0.06)	.68	1.06 (0.94-1.19)
Receiving help with daily activities^f^, column (%)		127 (7.3)	76 (7.6)	.80	1.18 (0.76-1.82)
Dementia, column (%)		43 (1.8)	15 (1.6)	.70	1.01 (0.50-2.04)
Living in a nonmetropolitan county, column (%)		313 (18.3)	108 (11.5)	.01	0.82 (0.53-1.28)
**Social deprivation index^g^**					
	Lowest tertile (<28), column (%)	586 (37.7)	393 (49.6)	<.001	Reference
	Top 2 tertiles (>28), column (%)	1022 (62.3)	436 (50.4)	N/A	0.87 (0.67-1.13)
**Broadband subscription^h^**					
	Lowest tertile (<60.9%), column (%)	465 (26.4)	153 (16.1)	<.001	0.61 (0.42-0.87)
	Middle tertile (60.9%-76.0%), column (%)	552 (35.3)	250 (30.2)	N/A	0.77 (0.58-1.03)
	Highest tertile (>76.0%), column (%)	591 (38.3)	426 (53.7)	N/A	Reference

^a^Weighted estimates of the 2017 National Health and Aging Trends Study (round 7): 13.5 million nonusers and 7.5 million users.

^b^Pearson chi-square test for frequencies and the adjusted Wald test for means.

^c^Adjusted odds ratios derived from a multivariable logistic regression model that is inclusive of all measures presented in the table.

^d^N/A: not applicable.

^e^Self-reported hearing difficulty, vision difficulty, heart attack, heart disease, hypertension, stroke, hip fracture, diabetes, cancer, lung disease, and arthritis.

^f^Daily activities include eating, bathing, toileting, and dressing.

^g^2015 social deprivation index tertile: ranges from 0 to 100; higher values indicate greater social deprivation.

^h^Broadband subscription rates among older adults in accordance with the 2017 American Community Survey (Table S2802).

In a multivariable logistic regression model, age, education, income, and neighborhood broadband subscription remained highly associated with older adults’ engagement in telehealth (Table 2): living in a neighborhood at the lowest (vs highest) tertile of broadband internet subscription was associated with a 40% lesser likelihood to engage in telehealth (adjusted odds ratio 0.61, 95% CI 0.42-0.87). No differences in telehealth use were observed by older adults’ gender, race, health, neighborhood social deprivation, or county metropolitan status in the regression model.

## Discussion

This nationwide study establishes that among community-living older adults who use the internet, the pervasiveness of neighborhood broadband internet is highly associated with engagement in telehealth. Prior studies have reported that more than 1 in 4 Medicare beneficiaries—more often those who are older, with lower incomes, and of Black or Hispanic racial or ethnic status—lack internet access at home [4,7] and that having more limited social and economic resources and living in a nonmetropolitan county are associated with “unreadiness” to engage in telehealth [2]. Our study extends this knowledge by drawing on a unique data source that affords the ability to differentiate older adults’ internet use and engagement in telehealth alongside comprehensive information on both individual and neighborhood characteristics. Our findings indicate that, all else equal, living in a neighborhood at the lowest (vs highest) tertile of broadband internet subscription is associated with a 40% lesser likelihood to engage in telehealth. Importantly, of those characteristics that were most highly associated with engagement in telehealth (including age, education, and income), neighborhood broadband subscription stands out as uniquely amenable to policy intervention.

A recent study reported that nearly half of all fee-for-service primary care visits occurred through telehealth following the COVID-19 outbreak and that telehealth usage was disproportionately concentrated in urban and metropolitan counties [1]. Our study confirms that prior to the emergence of the COVID-19 outbreak, among internet users, living in a metropolitan county and low neighborhood social deprivation are associated with engagement in telehealth. However, there is promise in our finding that the strengths of these associations are attenuated and not significant in the context of individual and regional characteristics, including the prevalence of neighborhood broadband subscription. Although the lack of broadband internet may compound other barriers to health care access—for example, more limited availability of health care professionals and poor transportation infrastructure in rural areas [5]—prior studies of barriers to telehealth use among older adults have primarily focused on individual attitudes, capabilities, and resources [15]. While the expansion of broadband availability would remove a significant barrier to telehealth access, expansion of telehealth use will require attention to a broader set of cost and access factors such as affordability of broadband services and computer equipment, as well as education and assistance with technologies to overcome gaps in technology experience [2,6].

This cross-sectional study does not provide insight into the frequency, quality, modality, or setting of telehealth interactions. The survey information analyzed in this study was from 2017, prior to the more flexible telehealth environment promoted by Centers for Medicare & Medicaid Services since March 2020. Our study results are limited to telehealth use among the subset of older adults who reported using the internet in the previous 30 days. Audio-only telephone-based telehealth communication, which has become common during the COVID-19 emergency [16] was not measured in this study. Despite these limitations, our findings reinforce the relevance of broadband internet as a consequential factor in equitable access to telehealth and suggest possibilities from policies and programs to extend broadband internet availability and affordability [17,18]. The COVID-19 outbreak has disrupted a wide range of health-producing activities including education, health care, and commerce. In this context, study findings resonate with calls to recognize and invest in broadband internet as a social determinant of health, which is especially significant during this time of heavy reliance on remote technologies [18].

## Abbreviations

NHATS: National Health and Aging Trends Study
